# Prevention and Treatment of CKD-MBD

**DOI:** 10.5812/numonthly.9372

**Published:** 2013-03-30

**Authors:** Mario Cozzolino

**Affiliations:** 1 Laboratory of Experimental Nephrology, Renal Division, University of Milan, San Paolo Hospital, Milan, Italy

**Keywords:** Vitamin D, Vascular Calcification, Renal Insufficiency, Chronic

Dear Editor,

Premature arterial ageing has been widely investigated because of its great impact on the general population and especially in chronic kidney disease (CKD) patients. Accelerated atherosclerosis and vascular calcification (VC) is the main histological manifestations of arterial ageing, characterized by cellular necrosis, inflammation, and lipid deposition (1). Differences between VC in general population and CKD patients are not only related to the type and localization of lesions, but also to the early age at which VC lesions begin in CKD patients (2). Common risk factors such as hypertension, ageing, smoking, diabetes, and dyslipidemia do not fully explain the high frequency of cardiovascular (CV) disease, suggesting the presence of other different pathological pathways (3). Moreover, vascular calcification in CKD could be no longer considered only a passive process resulting from an elevated calcium-phosphate product (4). In addition, both oxidative stress and inflammation are involved in vascular ageing (5). According to recent studies, vitamin D receptor activators (VDRAs) play an important role in the prevention of premature vascular ageing (6). The large distribution of VDRs suggests they are involved in different organ functions including cardiovascular, immune, gastrointestinal, and endocrine systems, considering some of these effects independent of calcium-phosphorus metabolism (6, 7). Studies on role of VDRs in premature aging are limited in humans, but large amount of data are known in experimental studies in animals. There are several preclinical and clinical studies investigating the mechanisms of ‘non-classical effects’ of vitamin D therapy, particularly on the cardiovascular system.

“CKD mineral bone disorders” (CKD-MBD) is a recent definition for abnormalities of bone mineral tissue, calcium-phosphorus metabolism, and VC. Mineral disorders in CKD typically consist of hypocalcemia, hyperphosphatemia, alterations of vitamin D metabolism, and secondary hyperparathyroidism (8). In addition, CKD-MBD is characterized by abnormalities of bone turnover, mineralization, volume and growth as well as VC. Indeed, the co-localization of bone markers such as osteopontin, alkaline phosphatase and osteocalcin along with osteoblast-like cells in the arterial wall of uremic patients indicate that VC is an active biological process and represents a possible link between calcium-phosphorus imbalance and the increased mortality associated with CKD-MBD (9).

In their manuscript, Seck et al. (10) showed the prevalence of CKD-MBD in three Dialysis Units in Senegal. Interestingly, high levels of PTH and high bone turnover was the most common CKD-MBD clinical feature of these patients, with almost 25% of HD patients affected by either vascular or valvular calcifications. Furthermore, management of CKD-MBD included phosphate binders (both calcium-based and calcium-free), vitamin D, and cinacalcet, even if treatment of this dramatic complication of CKD remains suboptimal for most patients.

CV disease is the major cause of death in general population and in CKD patients. Development of secondary hyperparathyroidism (SHPT) due to vitamin D deficiency and decreased VDR activation contributes to CV morbidity and mortality in CKD patients (11). CV effects include hypertension, vascular calcification, smooth muscle cell proliferation, and fibrosis, which lead to myocardial and arterial thickening, and left ventricular hypertrophy (12). Atherosclerosis and calcification are the main histological features of arterial ageing, whose manifestation is the deposition of a well-organised plaque.

The link between vitamin D deficiency and CV morbidity and mortality is currently arousing great interest. In CKD patients, this association is even stronger, because VDR activation decreases as a result of progressive renal impairment.

Furthermore, in CKD atherosclerosis and VC are accelerated, at least in part because of an impairment of VDR activation, together with higher P levels. In the clinical setting, there are also several FGF23 actions that need be considered. In this sense, increased knowledge of CKD-MBD may be useful to improve diagnostics and select future treatments. The discovery of FGF23 represents a novel factor in the pathogenesis of P handling in cardiovascular, bone and renal disease. The correct use of P binders and VDR activators may play a role in preventing cardiovascular disease and arterial ageing in CKD.
